# Cerebral angiitis associated with subarachnoid hemorrhage in Castleman’s disease: report of two cases

**DOI:** 10.1186/s12883-016-0585-4

**Published:** 2016-05-04

**Authors:** Jun Tanaka, Atsushi Fujita, Kohkichi Hosoda, Eiji Kohmura

**Affiliations:** Department of Neurosurgery, Kobe University Graduate School of Medicine, 7-5-1 Kusunoki-cho, Chuo-ku, Kobe, 650-0017 Japan

**Keywords:** Castleman’s disease, Subarachnoid hemorrhage, Cerebral angiitis, Interleukin-6

## Abstract

**Background:**

Multicentric Castleman’s disease (MCD) is characterized by a systemic lymphoproliferative disorder affecting systemic lymph nodes. Cerebrovascular involvements have rarely been reported, and to our knowledge, cerebral angiitis causing subarachnoid hemorrhage (SAH) in patients with Multicentric Castleman’s disease (MCD) has not been previously described.

**Case presentation:**

We identified two cases of MCD with SAH who were receiving immunosuppressive therapy with low dose prednisolone. Both patients presented with sudden-onset headache and were diagnosed with cortical SAH in the sulci by a computed tomography scan. Digital subtraction angiography showed segmental stenosis in the peripheral area of the middle cerebral artery. In both cases, cerebral angiitis causing SAH induced by a systemic inflammatory condition and elevated levels of interleukin (IL) -6 were suspected and resolved over a period of several months.

**Conclusion:**

Our cases highlight the clinical diversity of the potential causes of cerebral angiitis and expand the association of MCD and cortical SAH; however, cortical SAH patients have a more favorable outcome than aneurysmal SAH patients.

## Background

Castleman’s disease (CD) was initially described by Benjamin Castleman et al. in 1956 as a disease with solitary hyperplastic mediastinal lymph nodes containing small, hyalinized follicles and a marked interfollicular vascular proliferation [1]. CD is caused by infection of human herpesvirus 8 (HHV-8) and human immunodeficiency virus (HIV). Such viral infections cause B cells in the lymph nodes to excessively release interleukin (IL) -6 or similar polypeptides and cause several autoimmune clinical symptoms [2]. CD is classified into two subgroups, unicentric Castleman’s disease (UCD) and multicentric Castleman’s disease (MCD). UCD usually affects one lymph node area and the patients are often asymptomatic. In contrast, MCD affects systemic lymph nodes and the patients present various symptoms, such as hyperthermia, lymphadenopathy, splenomegaly, hepatomegaly, pulmonary disorders, edema, and ascites. MCD also may be associated with a variety of malignant diseases, such as Kaposi sarcoma, non-Hodgkin lymphoma, Hodgkin lymphoma, POEMS syndrome: polyneuropathy, organomegaly, endoclinopathy, monoclonal proteinemia and skin changes [2]. About the vascular involvements of MCD, temporal arteritis [3] and some cases of coronary and lower limb vascular involvements have been described. In general, it is unusual that the patients of MCD will present neurological symptoms, and intracranial involvements of the MCD have been rarely reported in the literature to date [4]. An association of MCD with some types of intracranial tumors, such as chordoid meningioma and clear cell meningioma, has been reported in some cases [5]. The pathogenesis of MCD in cases with such intracranial tumors remains unclear, but a complex cytokine network, including IL-6, IL-1β and vascular endothelial growth factor (VEGF), may contribute to the growth of the tumor [6]. Although there are some reports of cerebrovascular involvements in patients with MCD, all of the presented cases had ischemic stroke caused by cerebral angiitis secondary to a systemic inflammatory condition, as indicated by excessive serum levels of IL-6 [7, 8]. To our knowledge, no reports are currently available about cerebral angiitis causing subarachnoid hemorrhage (SAH) in patients with MCD. Here, we describe two rare cases of MCD associated with cerebral angiitis leading to SAH.

## Case presentation

### Case 1

**Fig. 1:**
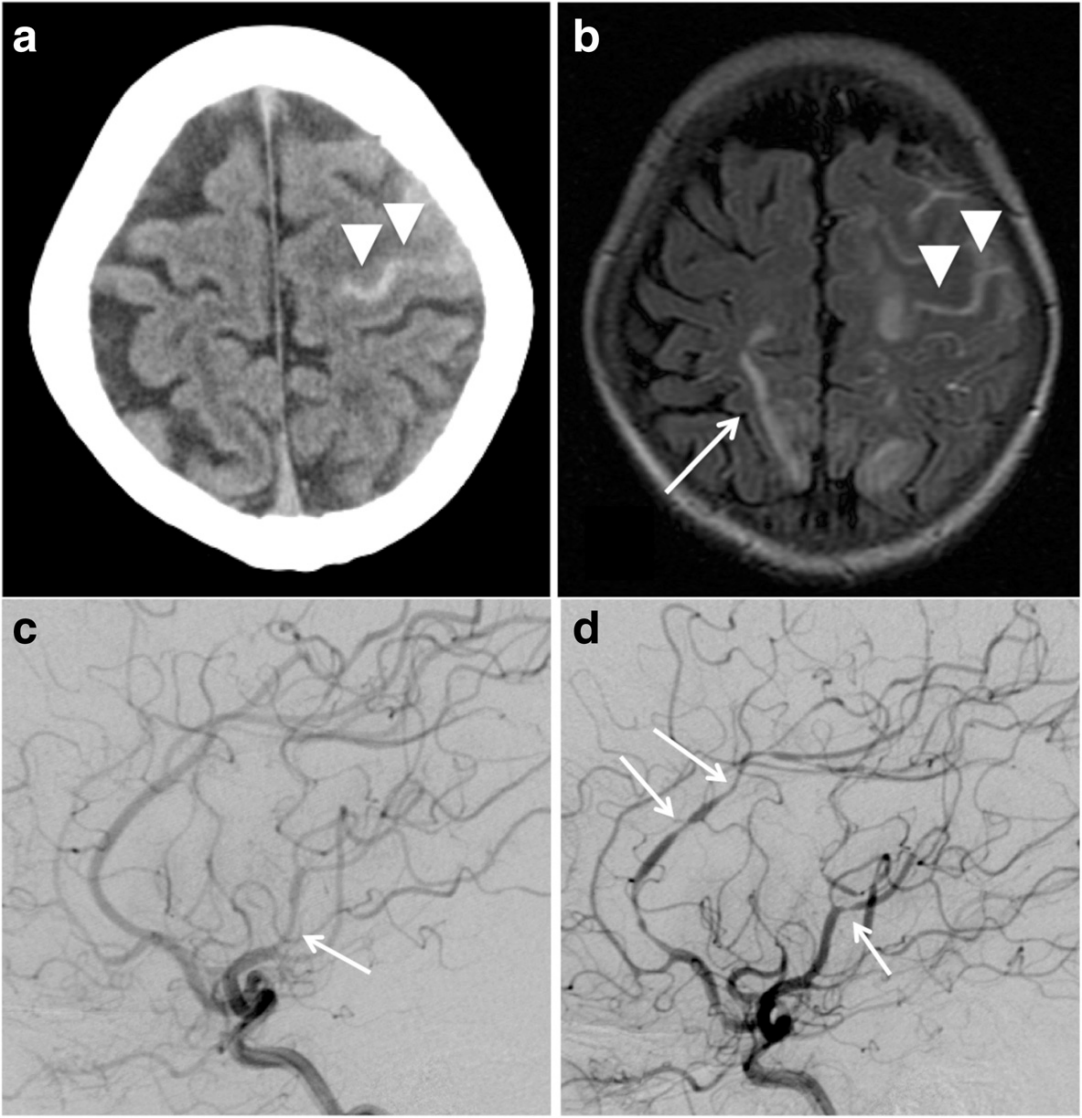
Neuroimaging findings of Case 1. **a** CT scan of the head shows a cortical subarachnoid hemorrhage along the left frontal sulci (*arrow heads*). **b** FLAIR sequence of the MRI shows the distribution of a subarachnoid hemorrhage (*arrow heads*) and hyperintense signal in the cortical-subcortical regions of the right parietal lobe (*arrow*). **c** Lateral view of the left internal carotid angiogram shows stenosis in the area of the peripheral middle cerebral artery. **d** Lateral view of the left internal carotid angiogram obtained ten days after onset shows diffuse segmental stenosis in the area of both the anterior and middle cerebral artery

A 57-year-old woman was admitted to a nearby hospital because of respiratory discomfort due to exercise, where a laboratory examination showed high levels of serum gamma globulin. She was then referred to our hospital for further examination. On admission, her physical examination showed a swelling of the lymph nodes of the mediastinum and axillary fossa. Laboratory examination showed anemia (hemoglobin: 3.9 g/dl), hypoalubuminemia (1.5 g/dl), hypergammaglobulinemia (IgG: 8140 mg/dl, normal: 870–1700 mg/dl, IgA: 851 mg/dl, normal: 110–410 mg/dl, IgM: 297 mg/dl, normal: 46–260 mg/dl) and an elevation of serum levels of C-reactive protein (CRP) (9.8 mg/dl). The serum IL-6 level was 43.5 pg/ml (normal < 4.0 pg/ml). An HIV viral examination was negative. HHV-8 examination was undone. A chest X-ray showed a reticular shadow of bilateral lung fields. Histopathological examination by transbronchial lung biopsy (TBLB) to lung lesions showed plasmacystic proliferation. The histopathological findings led to her to be diagnosed as MCD, and we started to administrate prednisolone orally (50 mg/day). Thrombocytopenia or coagulopathy (including anticoagulant-induced) was not identified based on the laboratory findings, and any other infection or autoimmune disorder was excluded. Three months after admission to our hospital, she suddenly felt a strong headache. She was alert and presented no apparent neurological deficit. The level of immunoglobulins and other laboratory findings were similar to those recorded previously. Her blood pressure was 148/88 mmHg, and her other vital signs were within normal range. A head computed tomography (CT) scan showed cortical SAH along the left frontal sulci (Fig. 1a). Further evaluation by fluid-attenuated inversion recovery (FLAIR) sequence of magnetic resonance imaging (MRI) showed the distribution of SAH and a hyperintense signal in the cortical-subcortical regions of the right parietal lobe (Fig. 1b). The corresponding diffusion-weighted images (not shown) were normal. We performed digital subtraction angiography (DSA) emergently to search for the source of bleeding. We could not find vascular abnormalities, but we did find segmental stenosis and delayed flow of the left middle cerebral artery (MCA) (Fig. 1c). We diagnosed that the stenosis due to angiitis caused both SAH and ischemic change, and we performed antihypertensive therapy by infusion of a calcium-channel blocker. The follow-up DSA obtained after ten days from onset showed newly segmental stenosis of the anterior cerebral artery (ACA) and progression of the stenosis of the MCA (Fig. 1d). Angiography of the bilateral renal artery in the same examination also showed stenosis of the peripheral renal artery (not shown). These results indicated that systemic angiitis associated with MCD might have occurred, and we continued prednisolone therapy (decreased to 22.5 mg/day). Fortunately, she did not present neurological focal deficit including high function disorder, and her headache became weakened day by day. SAH completely disappeared and no de novo stenosis of intracranial artery in the other portion by 1 month, as determined by MRI, and no other event was recognized during an 8-year follow-up period.

### Case 2

**Fig. 2:**
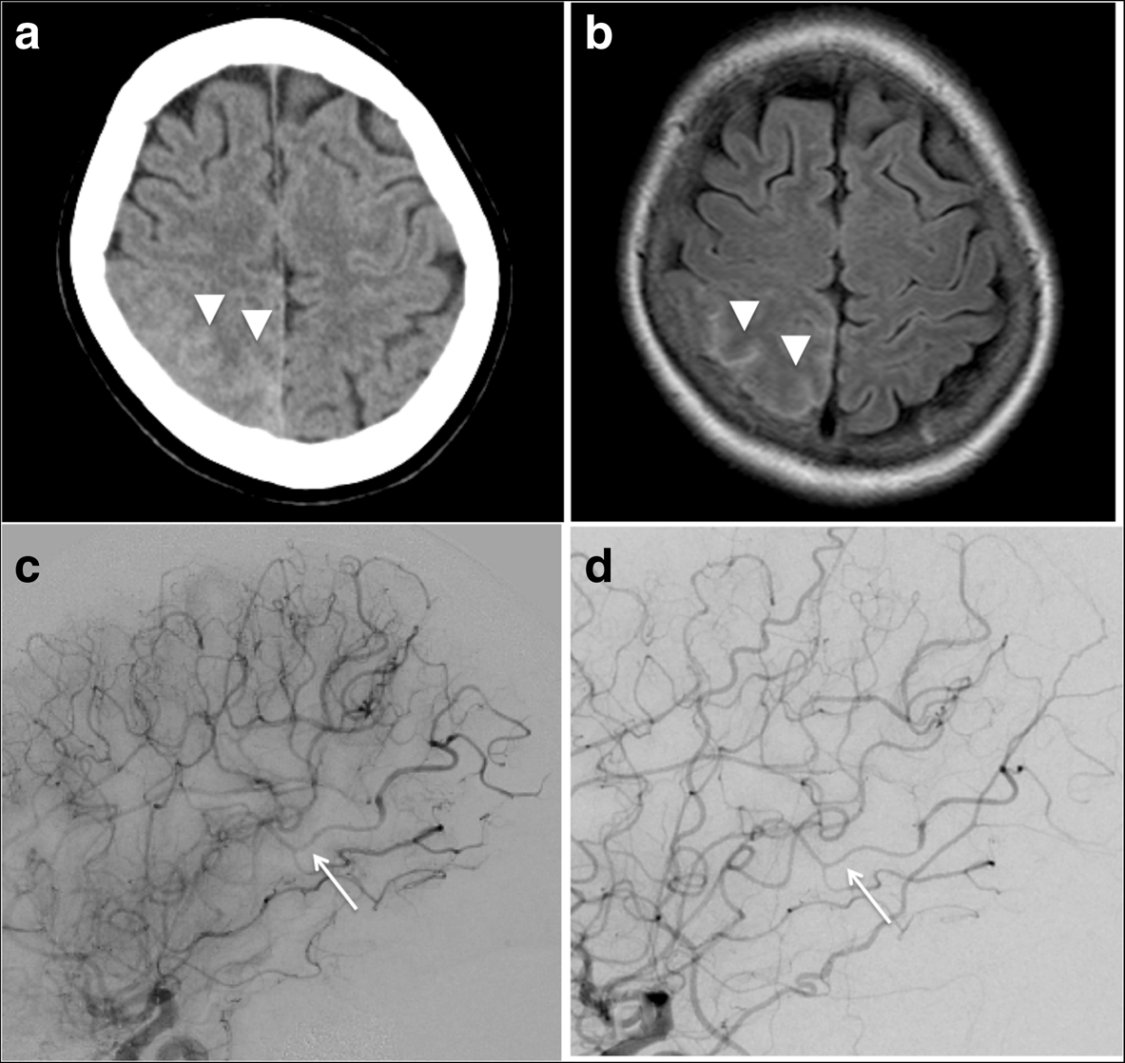
Neuroimaging findings of Case 2. **a** CT scan of the head shows a subtle cortical subarachnoid hemorrhage along the right parietal sulci (*arrow heads*). **b** FLAIR sequence of the MRI shows the distribution of a subarachnoid hemorrhage (*arrow heads*). **c** Lateral magnified view of the right internal carotid angiogram, late arterial phase, shows a stenosis (*arrow*) and post-stenotic delayed opacification in the area of peripheral middle cerebral artery. **d** Lateral magnified view of the right internal carotid angiogram obtained one week after onset shows a disappearance of stenotic findings and normal perfusion (*arrow*)

A 59-year-old woman found a rash on her abdomen 20 years ago. At 51 years of age, she was diagnosed with anemia and hypergammaglobulinemia at an annual health checkup. For further examination, she was admitted to a general hospital near her home. There, a physical examination did not find swelling of lymph nodes or any other abnormal findings. A laboratory examination found anemia (hemoglobin: 9.1 g/dl), hypoalubuminemia (2.1 g/dl), hypergammaglobulinemia (IgG: 6627 mg/dl, IgA: 798 mg/dl, IgM: 765 mg/dl) and an elevation of serum levels of CRP (9.8 mg/dl) and the serum IL-6 level (131 pg/ml). HIV viral examination was negative. HHV-8 examination was undone. Past examinations showed that she had neither thrombocytopenia nor coagulopathy. A chest and abdominal CT scan showed an interstitial shadow at the bilateral lung fields and mild splenomegaly. Biopsy of the lesion showed the proliferation of plasmacyte around vessels within true skin. She was diagnosed with MCD at 57 years old and started oral prednisolone (20 mg/day). At 58 years of age, she was introduced to our hospital because she moved to a home and continued prednisolone therapy. The condition of the disease was relatively stable, but the long-term administration of prednisolone was expected to risk systematic complications. We started to infuse tocilizumab, a humanized anti-human IL-6 receptor antibody, and decreased the dose of prednisolone to 12.5 mg/day. One day, she was admitted to the emergency room of our hospital complaining of sudden-on-set headache, and she was alert and presented no apparent neurological deficit. Her blood pressure was relatively high (180/90 mmHg), and other laboratory findings, including the level of immunoglobulin E, were almost within normal ranges. Head CT showed subtle cortical SAH along the right parietal sulci (Fig. 2a). FLAIR images showed the distribution of cortical SAH in right parietal sulci (Fig. 2b). The emergent right internal cerebral angiography, late arterial phase, showed stenosis and post-stenotic delayed opacification in the area of the peripheral middle cerebral artery; this perfusion area corresponded to the location of SAH (Fig. 2c). The follow-up DSA after one week after onset showed the disappearance of both stenosis and disturbed perfusion (Fig. 2d), and her headache got better and also the focal deficit was not presented. After 1-year from the discharge, cortical SAH was completely disappeared and no other vascular lesion was detected by the follow-up MRI, and no other event was recognized in the follow-up period.

## Discussion

Only in some cases in MCD, the cerebrovascular involvements of ischemic stroke due to cerebral angiitis have been reported, especially in the cases of MCD with POEMS syndrome [7, 8]. Rössler M et al. reported on a case of young female with POEMS syndrome presented recurrent ischemic stroke due to cerebral vasculitis. In this case, DSA showed the segmental stenosis of proximal ACA [9]. Garcia T et al. described two cases of POEMS syndrome and MCD accompanied with recurrent ischemic stroke, in which DSA showed diffuse non-atherosclerotic segmental stenosis of the intracranial arteries. They hypothesized that the stenosis might be induced by the overproduction of cytokines, such as IL-6, IL-1β and VEGF in MCD, however they concluded the pathogenesis remained unclear [10]. Among these inflammatory biomarkers, IL-6 is well known to play an important role in acute inflammatory response triggered by cerebral ischemia, and an elevated blood IL-6 concentration is associated with poor outcome after stroke [11]. Moreover, a higher level of serum IL-6 is also associated with an increased risk of recurrent vascular events after stroke [9]. Despite these conclusive findings in both acute and chronic phases that clearly indicate an important role of IL-6 in stroke patients, these associations are not fully understood. According to these reports, elevated levels of IL-6 might be a major causal factor in the occurrence of cerebral angiitis in our patients with MCD and a lasting chronic over-expression of IL-6. Further research is required to substantiate the role of these biomarkers and to determine whether any of them may serve as a prognostic factor or therapeutic target to prevent cerebral angiitis in MCD.

On the other hand, there is no report of hemorrhagic stroke due to cerebral angiitis in patients with MCD. In our two cases of MCD accompanied with SAH, it is pathognomonic that both cases presented with cortical SAH isolated along sulci, and both cases are accompanied with the segmental stenosis of peripheral intracranial arteries. The stenosis in these cases may be due to angiitis and SAH induced by the vulnerability of the arterial wall. This hypothesis is supported by the fact that the angiographic findings of angiitis appeared not only in intracranial arteries but also in bilateral renal arteries in case 1. As previously reported, the prognosis of patients with cortical SAH carried a favorable outcome than that of aneurysmal SAH [12]. Despite the presence of severe vasoconstriction, ischemic stroke, and cortical SAH, our case also experienced a favorable outcome, so a noninvasive imaging work-up, such as CT angiography or MR angiogram, should be introduced after an initial DSA does not identify any other underlying etiology.

Because there is no conclusive test that can be used to diagnosis cerebral angiitis, it is crucial to exclude the many other conditions that can mimic the symptoms of cerebral angiitis. First, patients with clinical and imaging features, such as severe headache, variable neurological deficits, and reversible arterial abnormalities (dilatations and stenosis), can be separately recognized as the reversible cerebral vasoconstriction syndrome (RCVS). Approximately one-third of patients with RCVS develop ischemic stroke, lobar hemorrhage, or convexal SAH. RCVS-related SAH likely results from dynamic caliber changes affecting cortical surface vessels, and it is also usually restricted to 1 to 2 sulcal spaces [13]. In our cases, reversible imaging findings were compatible with RCVS; however, we believe that the systemic inflammatory condition (i.e., MCD) possibly explained the reversible vasoconstriction. Second, the stenosis of intracranial arteries may be the ultra-early vasospasm that occurred immediately after SAH. Qurehi AI et al. reported in their study that 13 % of patients had evidence of ultra-early vasospasm (within the first 48 h), and the vasospasms were mild (vessel narrowing of 25–50 %) in 78 % of these patients [14]. Although immediate vasospasm of cortical SAH has been suspected, coexistence with these has never been reported in the literature. A possible explanation of this was that a small amount of extravascular blood of the cortical SAH has less local effect on cerebral arteries compared to aneurysmal one. Third, the long-term administration of glucocorticoid may also introduce the atherosclerotic stenosis of intracranial arteries. In such a case, the stenosis should not be segmental but widespread to all intracranial arteries, and we believe that it is difficult for the stenosis to recovery angiographically in such a short period. Moreover, the symptoms present relative slowly, not drastically. At present, we require additional careful considerations that distinguish RCVS or other possible causes from these systemic conditions until further studies can be performed.

## Conclusions

We identified two cases of MCD who presented with sudden-onset headache and were diagnosed with cortical SAH. In both cases, cerebral angiitis causing SAH induced by a systemic inflammatory condition due to MCD. Our cases highlight the clinical diversity of the potential causes of cerebral angiitis and expand the association of MCD and cortical SAH.

## Ethics and consent to participate

The authors declare that ethics approval was not required for this case report.

## Consent for publication

In case 1, written informed consent for publication of this case report and any accompanying images was obtained from the patient’s family because she had passed away. In case 2, written informed consent was also obtained from the patient. A copy of the written consent is available for review by the Editor-in-Chief of this journal.

## Availability of data and materials

The dataset supporting the conclusions of this article is included within the article.

## Abbreviations

ACA: anterior cerebral artery
CD: Castleman’s disease
CRP: C-reactive protein
CT: computed tomography
DSA: digital subtraction angiography
FLAIR: fluid-attenuated inversion recovery
HHV-8: human herpesvirus 8
HIV: human immunodeficiency virus
IL-6: interleukin-6
MCA: middle cerebral artery
MCD: multicentric Castleman’s disease
POEMS: polyneuropathy, organomegaly, endoclinopathy, monoclonal proteinemia and skin changes
RCVS: reversible cerebral vasoconstriction syndrome
SAH: subarachnoid hemorrhage
TBLB: transbronchial lung biopsy
UCD: unicentric Castleman’s disease
VEGF: vascular endothelial growth factor
